# Looking back at the diagnosis of PFAPA: a retrospecitve analysis of a prospective cohort study

**DOI:** 10.1186/1546-0096-13-S1-P49

**Published:** 2015-09-28

**Authors:** JS Hausmann, C Biggs, F Dedeoglu

**Affiliations:** 1 Boston Children's Hospital, Rheumatology, Boston, USA; 2 Beth Israel Deaconess Medical Center, Rheumatology, Boston, USA

## Introduction

Periodic fever, aphthous stomatitis, pharyngitis, and cervical adenitis (PFAPA) is the most common periodic fever syndrome of childhood. The original diagnostic criteria were introduced in 1989. However, published studies show large heterogeneity in PFAPA patients, promting attempts at refining the criteria.

## Objectives

To describe the clinical and laboratory findings in a large, prospective cohort of patients with PFAPA, to discuss the criteria used for diagnosis, and to revisit their original diagnosis.

## Methods

Children diagnosed with PFAPA were prospectively recruited from a large, tertiary hospital in Boston. Diagnosis was made by pediatric rheumatologists, otolaryngologists, or infectious diseases specialists with expertise in PFAPA. Clinical history was gathered at diagnosis, and laboratory testing was performed during flares. Years after diagnosis, we performed a retrospective chart review and reconsidered the original diagnosis of PFAPA.

## Results

76 patients were recruited; 70% were male. The ethnicity of the group was mixed, usually with more than one ancestry: 61% were Irish, 36% Italian, 26% English, 25% German, 22% Scottish, 20% French, 18% French Canadian, 11% Polish, 11% Portuguese, 9% Swedish, 7% Native American.

The average disease onset was 3 years of age. The frequency of cardinal features was as follows: 71% had pharyngitis, 67% had adenitis, 33% had aphthous stomatitis. 16% of childen had three cardinal features during flares, 50% had two features, 22% had one feature, and 12% had no features. In 66/68 (98%) patients, fevers occured at regular intervals. 14% of patients had disease onset at 5 years of age or older.

Prednisone was used at the onset of symptoms in 44 patients; 91% had complete response, 4% had incomplete response, and 1% did not respond.

In 46/48 (96%) of patients, inflammatory markers were elevated during flares. 26/44 (59%) had leukocytosis. 5/41 (12%) had lymphopenia,10/34 (29%) had eosinopenia, 17/37 (46%) had monocytosis.

10 patients had genetic testing for periodic fevers; 7 were negative, and one patient each had the following mutations: E148Q in MEFV, R92Q in TNFRSF1A, and PSTPIP1.

A retrospective chart review of these patients questioned the diagnosis of PFAPA in 27 (36%) patients due to lack of clear documentation of regular episodes of fevers (9), lack of any cardinal features (8), poor response to prednisone (1), follow-up notes revealed alternative diagnoses (eg hypogammaglobulinemia), or because the story did not seem consistent with PFAPA.

## Conclusions

Our study showed that physicians that frequently diagnose PFAPA do not use a common criteria for their patients. Only 16% of patients had the three cardinal features of PFAPA, while 12% had none. Leukocytosis was common during flares. Monocytosis, noted in prior studies, was seen in almost half of our patients.

However, almost all had evidence of systemic inflammation and showed resolution of the episodes with administration of prednisone. These features were not part of the original diagnostic criteria, but they may be useful for diagnosis.

